# Recent Advances and New Directions in Measuring Theory of Mind in Autistic Adults

**DOI:** 10.1007/s10803-018-3823-3

**Published:** 2018-12-04

**Authors:** Lucy Anne Livingston, Bethany Carr, Punit Shah

**Affiliations:** 1 Social, Genetic and Developmental Psychiatry Centre, Institute of Psychiatry, Psychology and Neuroscience, King’s College London, London, SE5 8AF UK; 2 Department of Psychology, University of Bath, Bath, BA2 7AY UK

**Keywords:** Theory of Mind, Autism spectrum disorder, Adults, Social cognition, Mentalising, Mindreading

## Abstract

‘Theory of Mind’ (ToM) is the ability to attribute mental states to others to make sense of their behaviour. ToM research has informed understanding of (a)typical social behaviour, including the symptoms of autism spectrum disorder (ASD). This began with research on ToM in autistic children and there has been a noticeable increase in the study of ToM in autistic adults. However, methodological limitations in adult ToM research may be limiting its explanatory power of ASD symptoms and their management, therefore we discuss recent advances in measuring ToM aimed at addressing these issues. We also examine previously overlooked approaches and propose several new directions that have potential to improve the sensitivity, accuracy, and clinical utility of ToM measurement in autistic adulthood.

Autism spectrum disorder (ASD) is characterised by atypical social-communicative and restricted and repetitive behaviours (American Psychiatric Association 2013). The causes of autistic symptoms are wide-ranging, however several mechanisms associated with ‘Theory of Mind’ (ToM)—the ability to attribute mental states to others—are widely thought to be atypical in ASD (Cantio et al. 2016; Happé et al. 2017). Atypical ToM is therefore a clinically relevant feature, and its accurate measurement is thought to be important for explaining and ameliorating social difficulties in ASD. Considerable research has focussed on ToM in autistic children (see Tager-Flusberg 2007), however there is less experimental research into, and critical discussion of, atypical ToM in adults. It is unclear if, or the mechanisms by which, atypical ToM contributes to the expression of symptoms and clinical outcomes in autistic adults (Jones et al. 2018). Moreover, given the growing rates of autistic children entering adulthood, individuals being diagnosed in adulthood, and therefore older adults with ASD, there is a need for more ToM research in autistic adulthood (see also, Lever and Geurts 2016).

## Measuring ToM in Autistic Adults

Conducting ToM research in autistic adults initially required changes to measures given to children that produced ceiling effects in adults (e.g., false belief tasks; Frith 1994). Therefore, ‘advanced’ measures were created to measure adults’ ability to reason about mental states in more complex contexts. The widely-used Strange Stories task (Happé 1994), for example, requires participants to infer false beliefs of characters in verbal vignettes, while the Reading the Mind in the Eyes Task (RMET; Baron-Cohen et al. 2001) requires matching static images of the eye with mental state words. These are considered ‘explicit’ measures as ToM is prompted by direct mental state questions. Despite their popularity and usefulness in advancing autism research (e.g., genetic aetiology; Warrier et al. 2017), there is growing awareness of the limitations of these ‘classical’ ToM tasks. IQ, for example, is highly correlated with performance on the Strange Stories task and the RMET (e.g., Baker et al. 2014). Accordingly, some individuals’ ToM ability is under-estimated due to verbal impairments, whereas individuals with good verbal skills may compensate for ToM impairments and perform well on these tasks (Livingston and Happé 2017). In addition, recent work challenges whether these tasks measure ToM or emotion processing, leading to caution against their use in autistic adults with emotional difficulties (Oakley et al. 2016; Olderbak et al. 2015). In attempts to address some of these issues, ‘implicit’ ToM tasks have been used to probe automatic behavioural responses to scenarios that induce spontaneous ToM (see Apperly and Butterfill 2009 for overview). These include measuring participants’ eye movements while they view scenarios where agents hold false beliefs (e.g., Senju et al. 2009) or the extent to which participants spontaneously attribute mental states to animated triangles (e.g., White et al. 2011). The theory and methodology surrounding ‘implicit’ ToM is, however, also not without controversy. Its existence and distinction from ‘explicit’ ToM is currently debated (Carruthers 2017), particularly given claims that many ‘implicit’ ToM tasks measure general attentional processing, not ToM (e.g., Heyes 2014; Santiesteban et al. 2015). Overall, therefore, studies using classical measures of ToM to measure ToM in autistic adults are inconsistent (see Chung et al. 2014, for meta-analysis).

We will not dwell on conceptual and theoretical debates about classical ToM tasks, which can be found elsewhere in the literature (see Brewer et al. 2017, for recent discussion). Indeed, we suggest that a focus on classical tasks, and resulting debate, is stymieing development of novel methods, thereby constraining the clinical relevance of ToM research in autistic adults. This is highlighted by several findings that individuals with ASD show relatively minor impairments on classical tasks compared with their difficulties observed in clinical settings and everyday social situations (e.g., Begeer et al. 2010; Couture et al. 2010; Lever and Geurts 2016; Scheeren et al. 2013; Schneider et al. 2013; Spek et al. 2010; Wilson et al. 2014). This finding is often attributed to differences between laboratory and naturalistic settings, yet little is done to understand this discrepancy. Addressing this issue offers a good opportunity to study ToM using behavioural tasks with the aim of supporting autistic individuals with everyday social difficulties (see also, Fletcher-Watson et al. 2014). To this end, we direct the readers’ attention to recent developments in measuring ToM that have particularly clear potential for improving understanding and management of ASD.

## Recent Advances

First, a computerised paradigm has been developed by Deschrijver et al. (2016) in which adult participants are required to track an agent’s belief about the location of a ball to measure ToM ability. Using a keypad, they are tasked with responding when the ball is revealed from behind an occluder. Critically, using the occluder, the ball’s actual location and the agent’s belief about its location are manipulated, so that the agent may hold a false belief about its location. Autistic adults, compared to matched controls, were slower to detect the ball when the agent falsely believed the ball was behind the occluder and the participant knew it was not. The group difference was not statistically significant, however performance on the task was closely associated with self-reported autistic traits and observational measures of autistic behaviour. Deschrijver et al.’s task is an ‘implicit’ measure of ToM, therefore it may be subject to the ongoing limitations and debate on the existence and measurement of ‘implicit’ ToM (see Nijhof et al. 2016). Nonetheless, this recent development indicates that ToM atypicalities, when measured by response time (RT) on appropriate cognitive tasks, may help to understand autistic symptoms in adulthood. More broadly, this suggests that measuring RTs—a relatively underused technique within classical ToM research—may be used to refine older ToM tasks, where RTs were previously not measured (see below).

Second, recently developed tasks have sought to measure ToM by examining accuracy, via open-ended or multiple-choice questions, of mental state attribution after watching video-clips of social situations (Brewer et al. 2017; Dziobek et al. 2006; Murray et al. 2017). Video-based tasks require more than simple inference from images or vignettes, and instead require inference of multiple socially relevant cues. This is designed to reflect mental state inference in ‘real’ social situations. Importantly, these tasks include non-ToM questions, allowing for alternative explanations of ToM impairment to be explored (e.g., poor memory). Although development of ToM videos may seem relatively straightforward, this process has been informed by extensive theoretical research and technological advancements. The advantage of these ecologically valid video-based tasks is demonstrated by the clarity of findings from recent studies (e.g., Murray et al. 2017), which report that autistic adults show impaired accuracy compared to neurotypical controls. Such clear difficulties on video-based tasks contrast with findings that autistic individuals perform well on classical ToM tasks (e.g., Chung et al. 2014).

Together, recent research demonstrates that new RT and video-based tasks can successfully measure atypical ToM. Large group differences between adults with and without ASD are being observed, far exceeding those found in previous research. Importantly, these results hold even after controlling for other cognitive abilities and appear to be consistent across studies. Furthermore, and importantly, recently developed ToM measures are sensitive to individual differences *within* samples of (a)typical adults, enabling their use in correlational analyses with other cognitive/behavioural processes associated with ToM (e.g., Shah et al. 2017). Most of these tasks are freely available, generating new opportunities and challenges to be addressed in future. Building on these advances, we propose a series of novel and previously overlooked approaches could further improve the measurement of ToM in research and clinical settings.

## New Directions

### Abbreviated Tasks

Video-based and RT tasks are more time consuming to administer than classical ToM tasks. Although not prohibitively long for use in small-scale studies, this may preclude their use in larger cohort studies and clinical practice. Shorter tools will, for example, be required in longitudinal studies measuring ToM across adulthood. We therefore suggest that researchers should abbreviate existing, or develop novel, ToM tasks. Importantly, this process should be undertaken carefully, ensuring that construct validity and psychometric properties are not sacrificed. There is a tendency to shorten tools without explanation, such as randomly removing questions from established autism questionnaires for use in genetic research (e.g., Taylor et al. 2015). To avoid these issues, it is suggested that psychometric analyses (e.g., factor structure, item analysis, internal consistency) are reported in detail when creating new, and abbreviating existing, ToM tasks. Abbreviated tasks should also be validated against existing tasks as part of their development. This process will benefit from collaboration between psychologists, non-psychologists (e.g., geneticists), clinicians, and even employers, who require shorter measures for practical reasons. It will also profit from ‘Patient and Public Involvement’ (Brett et al. 2014). For example, autistic adults’ input may be valuable when designing the content and duration of tasks to improve participant engagement when used in future research and clinical practice. Finally, their development will advance by improving scoring systems, whereby task performance can be calculated quickly, particularly outside of research contexts.

### Multiple Choice and Automated Scoring

In addition to time taken to administer video-based measures of ToM, it is challenging that, apart from the Movie for the Assessment of Social Cognition (Dziobek et al. 2006), they typically rely on coding of verbal responses (e.g., Murray et al. 2017). This is not ideal in populations with social-communication difficulties. Neurotypical individuals rate autistic individuals less favourably than other neurotypical individuals after a single exposure, even without knowing their diagnostic status (Sasson et al. 2017), therefore it is possible that verbal responses from autistic individuals influence the scoring of their data. Even if coders are blind to the study’s aims, it seems likely that idiosyncratic responses may be coded as ‘autistic-like’ by a neurotypical coder. To begin addressing these issues, we suggest that researchers could improve scoring systems to measure, and examine the correspondence between, multiple-choice and verbal responses in ToM tasks. It would then be possible to examine the extent to which (if at all) there is bias in neurotypical coding of autistic responses. This should be relatively clear if ToM difficulties are more pronounced when analysing verbal compared to multiple-choice responses. More generally, coding verbal responses carries financial and time costs due to the need for audio transcription and multiple coders, whereas the use of (computerised) multiple-choice questions provides a more efficient way to measure ToM ability. Overall, the development and increased use of objective and automated scoring systems should provide a more robust, quicker, and cost-effective way, to administer and score ToM tasks.

### Response Time and Neuroscience Data

Most ToM measures used in ASD research have taken *accuracy* as ToM *ability* (e.g., number of trials where mental states are correctly inferred). Measuring accuracy alone, however, can lead to misunderstandings and missed opportunities when investigating ToM in ASD. For example, when high accuracy, i.e., ‘good’ ToM ability, is observed in autistic participants, this has sometimes lead to the conclusion that ToM ability is typical in this population (e.g., Scheeren et al. 2013). However, it is possible that some individuals use alternative, potentially slower, cognitive strategies to ‘compensate’ for poor ToM ability, thus appearing to perform well on ToM tasks (Livingston et al. 2018). A simple, and surprisingly unexplored, way to explore this further could involve measuring participants’ RTs in computerised ToM scenarios (e.g., Nijhof et al. 2016). Using inverse efficiency scoring (i.e., combining RT and accuracy data; Bruyer and Brysbaert 2011), yet to be employed in ToM research, may also be informative. This could improve both classical and video-based tasks, and thereby help to explain why autistic individuals experience difficulties in processing socially-relevant cues in ‘real-time’. RT data are also likely to benefit research on the efficacy of clinical interventions (e.g., social skills training; Kandalaft et al. 2013), given that measuring change in ToM should be more accurate as RT-based tasks are less susceptible to practice effects from repeated testing. Finally, video-based ToM tasks are yet to be used in neuro- imaging and -stimulation studies in autism which, if conducted, could significantly improve understanding of the neurocognitive features of adults with and without ASD (see Wade et al. 2018 for recent discussion). Combining RT-based tasks with neuro- imaging and -stimulation methods (e.g., Nijhof et al. 2018) will be particularly interesting for elucidating whether ‘good’ ToM ability, where observed in ASD, is a result of compensation at the neurocognitive level (Livingston and Happé 2017; Mundy 2018).

### Web-Based Tasks

Following recent calls for improving the reproducibility of (clinical) psychological science (see Tackett et al. 2017), advancing research on ToM in ASD will necessitate improved designs and larger sample sizes. This will be particularly important for increasing statistical power to examine individual differences in atypical ToM, and their associations with clinically relevant features of ASD. Importantly, the development of web-based ToM tasks will also allow researchers and clinicians to reach autistic individuals who are unable to visit laboratory and clinical settings. Adapting ToM tasks for web-based platforms will therefore enable researchers to collect much larger datasets to address outstanding questions about ASD. Such research will be challenging and require innovation to ensure that tasks can be administered remotely without diminishing their reliability and validity. There is evidence that complex experimental tasks on the web are ‘as good as the lab’ in neurotypical samples (see Germine et al. 2012), however similar research in autistic adults will generate new challenges (e.g., communicating instructions) that will need to be addressed in future research. Despite these challenges, web-based research in ASD will afford several new opportunities in line with other directions outlined in this paper. It will particularly facilitate the collection of RT data, and larger datasets, that will be suitable for automated scoring and processing through machine learning algorithms (e.g., *PredPsych*; Koul et al. 2017).

### Interactive Contexts and Virtual Reality

ToM research in ASD has primarily focused on measuring cognition in autistic adults to explain their social symptoms. Of course, however, social difficulties arise not only from atypical cognition of the autistic adult, but also the social skills of people they are interacting with (Schilbach et al. 2013). Neurotypical adults tend to judge autistic individuals as socially awkward and people they are unlikely to be friends with (Sasson et al. 2017), which interferes with social interactive processes between neurotypical and autistic adults. There is surprisingly little research on this topic, however recent work indicates that neurotypical adults are better at inferring mental states and emotional expressions of neurotypical compared to autistic individuals (Brewer et al. 2016; Edey et al. 2016), suggesting that neurotypical individuals could misinterpret autistic individuals’ mental states and social-emotional behaviours. Equally, autistic adults report better understanding the minds, and predicting behaviour, of other autistic compared to non-autistic people (Milton 2012). Together, this indicates that neither autistic nor neurotypical ToM ability is being fully explored, given the current reliance on tasks using neurotypically-derived mental states (e.g., in video-based tasks, actors and script writers are neurotypical). Moving forward, autistic adults’ ToM of other autistic minds and neurotypical ToM of autistic minds should be investigated further to explore (a) whether autistic ToM ability is better for other autistic compared to neurotypical minds, and (b) whether social difficulties in autism arise, in part, from neurotypical difficulties with understanding autistic minds. To this end, there is growing realisation that ToM measures should be improved to measure ToM within an interactive context (see Mundy 2018). Even video-based tasks, despite good ecological validity, only require participants to use ToM as an observer, and not as an active participant within an interaction. Measuring ToM during a realistic interaction will enable researchers to understand how autistic adults’ social difficulties vary with the complexity of the interaction and whom they are interacting with. This might be achieved by creating an adult version of a recently developed naturalistic reciprocal interactive task for autistic children (see van Ommeren et al. 2017), and taking advantage of technological developments in virtual reality (e.g., interactive social avatars) despite the fact there remain several difficulties of using these technologies in autism research (see Pan and Hamilton 2018 for a detailed review).

### Clinical Utility

Autistic adults are often asked to describe their social difficulties and clinicians perform observational assessments, but there has been little effort to directly measure social cognitive difficulties in clinical settings due to practical considerations. Shorter, automated, web-based tasks might ultimately provide a practical way for clinicians to administer ToM tasks using standard web-enabled devices (i.e., tablet PCs). Video-based ToM tasks may be especially useful, given they are more representative of social-communication in naturalistic settings. Following their abbreviation, and implementation on web-based platforms, they should be practical to administer remotely before the client attends a clinical session. Alternatively, they may be used during time-limited sessions, in which discussion of ToM videos may act as a useful aid for clinicians to gain further insight into their clients’ difficulties. The videos could provide a structured way to examine the reasons underlying autistic adults’ difficulties in social situations, with scope for the clinician to provide personalised cognitive strategies to help them deal with social situations outside the clinic. More broadly, the use of ToM tasks in the clinic will contribute to applied research on the discrepancies between cognitive features and behavioural symptoms of ASD (see Livingston and Happé 2017), therefore generating new directions for future clinical research and practice.

### Intact ToM and Basic ToM Research

It is important for researchers and clinicians to be open to the possibility that not all autistic individuals have atypical ToM and basic research on ToM requires improvement. There is currently no evidence that *all* autistic adults have impaired ToM, and improved research on the causes and consequences of ToM differences *within* the autistic population is likely to help explain the heterogenous presentation of ASD. Some individuals may genuinely experience mild difficulties, while other individuals’ impairments might only become apparent when social cues are ambiguous during rapidly changing social situations. Additionally, there may be individuals who, despite difficulties as children, improve their ToM moving into adulthood. As the issues discussed in this paper are addressed, and we become more confident that ToM tasks are successfully capturing subtle atypicalities, it will be important to continue investigating intact ToM where observed in ASD. Likewise, the concept of ToM, though well-established, and widely used in and outside of autism research, will necessarily evolve as larger, more diverse, datasets emerge from empirical studies in typical and atypical adults. We have steered away from longstanding philosophical and conceptual debates about the construct validity of ToM in the current paper, with the aim of focussing on practical ways to improve the clinical utility of ToM research in the near term. Of course, however, basic research on ToM will, particularly in the long term, inform how we conceptualise and measure mentalising processes in autistic adults (Schaafsma et al. 2015).

## Summary and Conclusion

Atypical ToM is a clinically relevant feature of ASD, however difficulties in measuring ToM in ASD has constrained understanding of, and support for, social-communication difficulties in autistic adults. Recent advances in the field have improved the measurement of ToM in adults with ASD, as evidenced by their atypical performance on RT- and video-based ToM tasks that is in line with their symptom severity. Moving forward, we suggest that these tasks are abbreviated, so that they are suitable for collecting large datasets on web-based platforms and useful in clinical settings. Future use of RT measurement should also improve task sensitivity and overcome issues of autistic adults using compensatory strategies to ‘solve’ ToM tasks. Other novel approaches, including investigation into neurotypical ToM of autistic minds and autistic ToM of other autistic minds, should facilitate the move toward a suitably nuanced understanding of ToM in ASD in the context of ‘real-world’ interactions. Together, these developments will enable researchers and clinicians to better understand why most autistic adults, despite performing well on some ToM tasks, experience profound social-communication difficulties. It is hoped this will inform future clinical practice to assist autistic adults to manage their condition.
